# Evidence-based Tool for Triggering School Closures during Influenza Outbreaks

**DOI:** 10.3201/eid1602.091628

**Published:** 2010-02

**Authors:** Richard L. Vogt

**Affiliations:** Tri-County Health Department, Greenwood Village, Colorado, USA[REMOVED HYPERLINK FIELD]

**Keywords:** Influenza A, subtype H1N1, pandemic, surveillance, influenza outbreak, school closure, viruses, expedited, Japan, letter

**To the Editor:** I read with interest the recent article by Sasaki et al., “Evidence-based Tool for Triggering School Closures during Influenza Outbreaks, Japan” (*1*), which describes an algorithm for determining the optimal timing of school closures to control influenza outbreaks. The published information is a helpful guide for predicting influenza outbreaks in school settings. However, no data are presented to show the efficacy of school closures after the detection of such outbreaks. As such, the title “Evidence-based Tool for Predicting Influenza Outbreaks, Japan” would more accurately describe the article.

The findings presented by Sasaki et al. (*1*) could be used to help make a decision for school closure or dismissal in places like Japan, but no information is provided on whether this approach is effective in preventing further influenza virus transmission. This is an important distinction and should not change the current school response guidance published by the Centers for Disease Control and Prevention (CDC) (*2*). In general, CDC guidance suggests that during an influenza outbreak, policymakers should weigh the advantages and disadvantages of school dismissals or school closures before making a decision.
