# Supplementary material for: Evidence-based Tool for Triggering School Closures during Influenza Outbreaks
Source: Emerg Infect Dis. 2010 Feb;16(2):367–8. doi: 10.3201/eid1602.091628 (PMC2958033; doi:10.3201/eid1602.091628)
Supplement: Related texts

# Letter

**DOI:** 10.3201/eid1602.091719

**Published:** 2010-02

**Authors:** Asami Sasaki, Anne Gatewood Hoen, John S. Brownstein

**Affiliations:** Department of Health and Nutrition, University of Niigata Prefecture, Niigata, Japan (A. Sasaki); Division of Emergency Medicine, Children’s Hospital, Boston, Massachusetts, USA (A.G. Hoen, J.S. Brownstein); Department of Pediatrics, Harvard Medical School, Boston (A.G. Hoen, J.S. Brownstein)

## In Response

Vogt (*1*) correctly points out that our article (*2*) did not present data on the effectiveness of school closures to control influenza outbreaks. However, public health agencies continue to support school closure as a nonpharmaceutical response to the ongoing outbreak of pandemic (H1N1) 2009 (*3*) despite little evidence for the appropriate timing of closures, even though it is known that timely action is critical. As the title of our article reflects, our algorithm was designed as an evidence-based tool for supporting the timing of school closures.

In our article, we pointed out that evaluating the impact of school closures is a critical research question. Before April 2009, decision-making regarding school closure in Japan was left to individual schools, 98% of which are public. Since then, recommendations for public school closure have been made according to standardized rules set by the Japanese School Health and Safety Law, leaving final decision-making authority up to local education boards. Our next study will evaluate the effectiveness of this early, standardized timing of school closure in Japan.

On September 24, 2009, the Japanese Ministry of Health, Labor and Welfare presented a school closure plan for use in the different stages of an influenza outbreak; the plan is based on World Health Organization recommendations (*3*). In the early periods of an outbreak, when infection is not widespread, a “proactive school closure plan” will be put into effect; school closure for 5–7 days will be implemented as soon as the first infection is detected in a school. If the outbreak is already spreading through the community, “reactive school closure” is considered sufficient. This closure has been performed during seasonal influenza outbreaks in Japan, closing classes when many students were absent from school. Most schools now close when >10% infection-related absenteeism is reached. Our study, which provides a refinement of thresholds predictive of serious outbreaks in schools, may support this plan in Japan and provide schools worldwide with an approach to considering the timing of school closures.

## References

[1] Vogt R. Evidence-based tool for triggering school closures during influenza outbreaks [letter]. Emerg Infect Dis. 2010;16:367.2011359310.3201/eid1602.091628PMC2958033

[2] Sasaki A, Gatewood Hoen A, Ozonoff A, Suzuki H, Tanabe N, Seki N, Evidence-based tool for triggering school closures during influenza outbreaks, Japan. Emerg Infect Dis. 2009;15:1841–3.1989188010.3201/eid1511.090798PMC2857248

[3] World Health Organization. Global Alert and Response (GAR). Measures in school settings; pandemic (H1N1) 2009 briefing note 10 [cited 2009 Dec 14]. http://www.who.int/csr/disease/swineflu/notes/h1n1_school_measures_20090911/en/index.htm**l**
